# Endometriosis-associated infertility: Multi-omics insights into pathogenesis and precision therapeutics

**DOI:** 10.3389/fendo.2025.1613334

**Published:** 2025-10-02

**Authors:** Yuyi Ou, Hao Wang, Cankun Zhou, Yonglian Chen, Jun Lyu, Minqing Feng, Xiaobin Huang

**Affiliations:** ^1^ Department of Gynecology, Foshan Women and Children Hospital Affiliated to Guangdong Medical University, Foshan, China; ^2^ Department of Clinical Research, First Affiliated Hospital of Jinan University, Guangzhou, China

**Keywords:** endometriosis, infertility, hormonal dysregulation, immune dysfunction, oxidative stress, microbiome, therapeutic strategies

## Abstract

**Introduction:**

Endometriosis is a prevalent, estrogen-dependent, inflammatory disease that impairs fertility via hormonal dysregulation, immune dysfunction, oxidative stress/ferroptosis, genetic and epigenetic alterations, and microbiome imbalance. We summarize multi-omics insights and clinical implications for endometriosis-associated infertility.

**Methods:**

This article is a Systematic Review that synthesizes recent multi-omics and clinical evidence on mechanisms (hormonal, immune-inflammatory, oxidative stress/ferroptosis, genetic/epigenetic, microbiome/metabolic) and appraises therapeutic strategies spanning surgery, hormonal suppression, assisted reproductive technologies (ART), and emerging adjuncts. Mechanistic and clinical findings are integrated to map targets, biomarkers, and precision-care opportunities across disease phenotypes.

**Results:**

Evidence indicates local estrogen dominance with progesterone resistance, pervasive immune dysregulation, and oxidative stress with iron-driven ferroptosis that particularly injures granulosa cells, alongside disease-relevant genetic/epigenetic regulators and reproductive-tract/gut microbiome dysbiosis. Clinically, endometriosis detrimentally affects ovarian reserve and oocyte competence, disrupts endometrial receptivity/decidualization, and remodels pelvic anatomy through adhesions and fibrosis, cumulatively reducing fecundity. Current management includes laparoscopic excision/ablation, hormonal suppression (e.g., progestins, GnRH analogs/antagonists), and ART tailored to goals and disease severity. Adjunctive antioxidant and immune-modulating approaches show promise but require robust clinical validation. Biomarker discovery—including epigenetic regulators and microbiome-derived signals—may enable earlier diagnosis and personalization. Innovative avenues include immunotherapy targeting nociceptor–immune crosstalk, ferroptosis modulation, microbiota manipulation, and diet-based metabolic strategies.

**Discussion:**

The pathogenesis of endometriosis-associated infertility is multifactorial and interconnected. While current treatments offer benefits, their efficacy is variable. The integration of multi-omics data is unveiling novel diagnostic biomarkers and therapeutic targets. Future management requires a patient-centered, multidisciplinary precision medicine approach that combines mechanistic insights with individualized treatment strategies to improve reproductive outcomes across the disease spectrum.

## 1 Introduction

Endometriosis is a chronic, inflammatory gynecological disease characterized by the presence of endometrial-like tissue outside the uterine cavity, primarily affecting women of reproductive age and manifesting with symptoms such as pelvic pain and infertility. It is a prevalent condition, impacting approximately 10% of women of reproductive age worldwide—equating to around 190 million individuals—and is highly associated with infertility, present in 30–50% of women seeking infertility evaluation (1, 2). Global Burden of Disease (GBD) studies reveal declining trends in endometriosis incidence and prevalence. From 1990 to 2019, China experienced a 30% reduction in age standardized incidence rate (ASIR), with an annual decrease of 1.2% (3). Similar global patterns showed annual declines of 0.21% in incidence and 0.29% in prevalence during 1990-2017 (4). Despite these reductions, endometriosis remains a leading cause of gynecological disability-adjusted life-years (DALYs), particularly in high-income regions (5).

The pathophysiology involves complex interactions of endocrine, immunologic, and inflammatory processes, with retrograde menstruation as the most accepted theory for ectopic tissue implantation (6).Estrogen metabolism and chronic inflammation are key pathophysiological mechanisms, with emerging evidence highlighting the microbiome—particularly the gut and genital tract microbiota—in modulating estrogen metabolism and inflammation, potentially influencing disease etiology and symptomatology (7). The disease impairs fertility through multiple mechanisms, including anatomical distortions, a hostile pelvic environment due to chronic inflammation and oxidative stress, poor oocyte quality, impaired folliculogenesis, reduced ovarian reserve, and altered endometrial receptivity (6). For instance, endometriosis fosters a pro-oxidative environment with increased oxidative stress, negatively impacting oocyte development and endometrial function. Fibrosis, characterized by abnormal extracellular matrix accumulation in ectopic lesions, contributes to infertility by causing pelvic adhesions and impairing reproductive organ function (8). Additionally, hormonal imbalances such as progesterone resistance may reduce endometrial receptivity to embryo implantation (9). Monthly fecundity rates in couples with endometriosis-associated infertility range from 2% to 10% (10), and spontaneous conception rates vary by disease severity, with approximately 50% of women with minimal/mild endometriosis conceiving without treatment, compared to 25% with moderate disease and few with severe disease (11).

Clinical presentation is heterogeneous, encompassing menstruation-related pain (dysmenorrhea, dyspareunia), noncyclic pelvic pain, and infertility, with pain resulting from high densities of sensory nerve fibers in lesions and inflammatory processes (12). These symptoms markedly reduce quality of life and are associated with stress-induced psychological sequelae that contribute to anxiety, depression, low self-esteem, and relationship strain. Notably, illness management is a dyadic phenomenon, requiring women and their partners to navigate infertility together, though their shared experiences remain underexplored. The population of infertile women with endometriosis is heterogeneous, with diverse phenotypes complicating diagnosis and mechanistic understanding (13).

Diagnosis remains challenging, often delayed by an average of 7 years from symptom onset, due to overlapping symptoms with other conditions and reliance on invasive laparoscopy with histological analysis as the gold standard (12). This delay may allow disease progression and worsen treatment responses. Societal burden is substantial, including economic costs estimated at $22 billion annually in the United States alone, alongside negative impacts on education, employment, and mental health. Despite affecting millions globally—with estimates of 176 million women worldwide—research funding remains insufficient relative to its socioeconomic impact, hindering advancements in diagnostics and therapeutics (14). Modern management emphasizes a patient-focused, multidisciplinary approach addressing pain, fertility, and overall well-being, considering stress, systemic comorbidities, and the need for long-term care. Given its chronic, progressive nature and strong association with infertility, endometriosis represents a critical clinical and societal challenge requiring integrated strategies to improve diagnosis, treatment, and support for affected individuals (15).

## 2 Pathophysiological mechanisms underlying infertility in endometriosis

**Figure 1:**
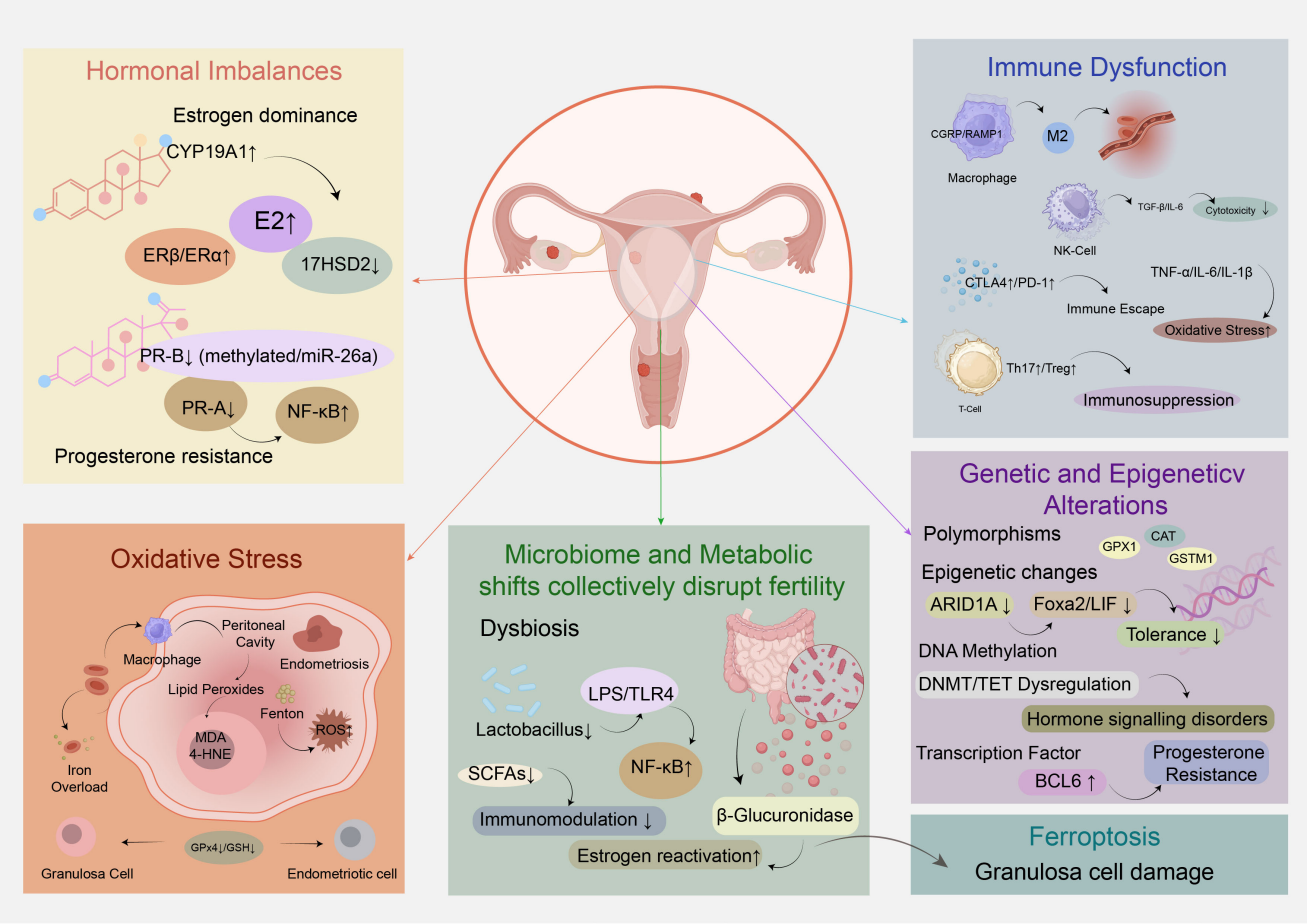
Molecular mechanisms underlying endometriosis-associated infertility.

Endometriosis-associated infertility remains a complex and challenging clinical issue, with its underlying pathophysiological mechanisms being multi-faceted and interconnected. Unraveling these mechanisms is crucial for developing targeted therapeutic strategies to improve reproductive outcomes in affected individuals. This section will delve into the key biological processes that drive infertility in endometriosis, exploring how hormonal imbalances, immune dysfunction, oxidative stress, genetic and epigenetic alterations, as well as microbiome and metabolic shifts collectively disrupt fertility (**Figure 1**).

### 2.1 Hormonal and endocrine dysregulation

Estrogen dependency and progesterone resistance are central to the hormonal and endocrine dysregulation driving endometriosis-associated infertility, facilitating ectopic endometrial implantation through impaired apoptosis, heightened inflammation, and aberrant neuroangiogenesis (6). Contrary to circulating estrogen levels, which are not significantly altered in endometriosis patients, local estrogen dominance arises from *de novo* synthesis within ectopic lesions and enhanced estrogen receptor signaling. Endometriotic tissue overexpresses aromatase (encoded by CYP19A1) and downregulates 17β-hydroxysteroid dehydrogenase type 2 (17HSD2), leading to increased estradiol (E2) production and reduced conversion to less potent estrone. Concurrently, an elevated ERβ/ERα ratio, resulting from promoter methylation-induced ERβ upregulation and ERα downregulation, amplifies estrogen signaling in endometriotic cells. Epigenetic modifications, including hypomethylation of ERβ and aromatase promoters, further sustain this estrogen-driven phenotype, with endocrine-disrupting chemicals (EDCs) such as dioxins and phthalates potentially exacerbating these epigenetic alterations (16).

Progesterone resistance, characterized by impaired progesterone receptor (PR) signaling despite bioavailable progesterone, perpetuates ectopic lesion survival and contributes to infertility (17). Endometriotic lesions exhibit marked reductions in PR-B isoform expression and decreased PR-A levels, attributed to promoter hypermethylation, microRNA dysregulation (e.g., miR-26a, miR-181), and genetic polymorphisms like PROGINS that disrupt ligand binding and downstream signaling. Functionally, progesterone fails to induce epithelial 17β-HSD2 in endometriotic tissue, sustaining high local E2 concentrations, and loses its ability to suppress NF-κB-mediated inflammation, thereby promoting lesion establishment and maintenance. This resistance undermines the efficacy of progestin therapies, necessitating prolonged hormonal suppression to reduce ectopic implant viability (18).

The interplay of estrogen dominance and progesterone resistance disrupts endometrial homeostasis, directly impacting fertility. Altered hormonal signaling impairs decidualization, disturbs the expression of implantation markers, and compromises embryo implantation in both eutopic endometrium and ectopic lesions. Additionally, estrogen-stimulated cyclooxygenase-2 (COX-2) activity drives prostaglandin E2 (PGE2) synthesis, creating a positive feedback loop that enhances local estrogen production and inflammation, further facilitating ectopic implantation (19). These mechanisms collectively highlight the rationale for hormonal therapies targeting estrogen suppression and overcoming progesterone resistance as strategies to improve fertility outcomes in endometriosis patients.

### 2.2 Immune system dysfunction and chronic inflammation

Immune system dysfunction and chronic inflammation are central pathological features underlying endometriosis-associated infertility, characterized by aberrant immune cell activation, cytokine dysregulation, and impaired immune surveillance. Endometriosis often coexists with adenomyosis, and both conditions share immunological alterations, including abnormal immune cell function and pro-inflammatory cytokine secretion, which collectively disrupt reproductive processes (20).

#### 2.2.1 Immune cell alterations

Macrophages are key drivers of immune dysfunction, constituting over 50% of immune cells in the peritoneal fluid of affected women. Neuroimmune communication via calcitonin gene-related peptide (CGRP) and its coreceptor RAMP1 promotes macrophage recruitment and phenotypic shifts toward a “pro-endometriosis” state, characterized by impaired efferocytosis and enhanced support of endometrial cell growth (21). This neuropeptide-mediated pathway operates independently of classic chemokine receptors like CCR2, directly stimulating macrophage secretion of chemokines and matrix metalloproteinases that facilitate lesion establishment. Additionally, peritoneal macrophages exhibit reduced phagocytic activity due to downregulated CD36 expression, allowing ectopic endometrial cells to evade clearance. Macrophage polarization is also dysregulated: women with endometriosis show M1 (pro-inflammatory) predominance in eutopic endometrium and M2 (anti-inflammatory/pro-angiogenic) polarization in ectopic lesions, supporting angiogenesis and tissue remodeling (22). Small extracellular vesicles (sEVs) derived from macrophages further modulate endometrial stromal cell behavior by transferring microRNAs and long non-coding RNAs, enhancing proliferation and invasion via SIRT1/NF-κB signaling (23).

Natural killer (NK) cell function is severely compromised, with reduced cytotoxicity of the CD56dimCD16+ subset in peripheral blood and peritoneal fluid, enabling immune escape of ectopic cells (24). This impairment is mediated by cytokines such as TGF-β, IL-6, and IL-15, which suppress NK cell activity (22).

T-cell subsets are dysregulated, with increased Th2, Th17, and regulatory T (Treg) cells in the peritoneal microenvironment (24). Th2 and Th17 cytokines promote inflammation and endometrial cell proliferation, while Tregs induce local immunosuppression by polarizing macrophages into pro-repair subtypes via soluble fibrinogen-like protein 2 (sFGL2) (23). A Th1/Th2 imbalance favoring Th1 predominance, associated with elevated TNF-α, IL-6, and IL-1β, further disrupts endometrial receptivity (25).Recent evidence further implicates neuroimmune crosstalk via CGRP/RAMP1 in macrophage polarization, as detailed in Section 6.1.

#### 2.2.2 Immune checkpoint molecules

Immune checkpoint pathways are perturbed, contributing to immunosuppression. CTLA4, a critical regulator of T-cell activation, is upregulated on CD4+ and CD8+ T cells in endometriosis, with elevated soluble CTLA4 (sCTLA4) in serum and peritoneal fluid of late-stage and infertility-associated cases. CTLA4 gene polymorphisms (+49A/G, CT60 A/G) are not linked to disease susceptibility, but experimental models show that CTLA4 blockade reduces ectopic lesion growth by inhibiting Treg-derived IL-10 and TGF-β (26). Programmed cell death protein 1 (PD-1) is also upregulated on T cells, induced by endometriosis stromal cells to facilitate immune evasion (24).

#### 2.2.3 Autoimmune overlaps

Endometriosis exhibits autoimmune-like features, including autoantibodies to endometrial antigens and polyclonal B-cell activation (10). Transcriptomic analysis identifies five autoimmune disease-related hub genes (CXCL12, PECAM1, NGF, CTGF, WNT5A) differentially expressed in ectopic *vs*. eutopic endometrium, with diagnostic value. Immune infiltrates in ectopic lesions include increased macrophages, mast cells, and memory CD4+ T cells, alongside decreased NK cells and plasma cells, mirroring autoimmune pathologies (27). These immune alterations parallel mechanisms in rheumatoid arthritis, where pro-inflammatory cytokines (TNF-α, IL-6) and immune cell dysfunction drive chronic inflammation (28).

#### 2.2.4 Chronic inflammatory milieu

Pro-inflammatory cytokines (TNF-α, IL-6, IL-1β, IL-8, IL-17) and chemokines (RANTES) accumulate in peritoneal and follicular fluid, creating a hostile microenvironment. TNF-α impairs endometrial receptivity, induces sperm apoptosis via caspase activation, and promotes ectopic adhesion through upregulation of ICAM1. Oxidative stress, generated by activated immune cells, produces reactive oxygen species (ROS) and nitric oxide, causing DNA damage in gametes and embryos, reducing sperm motility, and impairing fertilization (10). Endometriosis cells also exhibit malignancy-like properties, including invasiveness and responsiveness to TNF-α and COX-2, further exacerbating lesion persistence (29). Collectively, these immune and inflammatory perturbations disrupt folliculogenesis, ovulation, fertilization, and implantation, contributing to infertility.

### 2.3 Oxidative stress and ferroptosis in endometriosis

Ferroptosis, a novel iron-dependent programmed cell death characterized by the accumulation of lipid peroxides, is distinct from apoptosis, necrosis, and autophagy, and has been closely linked to female infertility, including endometriosis-associated infertility (30). A key hallmark of endometriosis is iron overload resulting from periodic bleeding in ectopic lesions. Retrograde menstruation transports erythrocytes into the peritoneal cavity, where macrophages actively participate in iron metabolism; iron released from erythrocyte hemolysis induces oxidative cellular damage and generates high levels of reactive oxygen species (ROS) (31). Excess iron catalyzes the Fenton reaction (Fe^2+^ + H_2_O_2_ → Fe^3+^ + OH^−^ + ·OH), producing highly toxic hydroxyl radicals that target lipids, proteins, and nucleic acids, thereby exacerbating oxidative stress.

In granulosa cells of women with endometriosis, elevated lipid peroxidation markers such as malondialdehyde (MDA) and 4-hydroxynonenal (4-HNE) have been observed, accompanied by decreased glutathione (GSH) and glutathione peroxidase 4 (GPx4), key inhibitors of ferroptosis (32). Mechanistically, ferritinophagy mediated by nuclear receptor coactivator 4 (NCOA4) promotes the release of labile iron in granulosa cells, enhancing lipid peroxidation via the Fenton reaction and facilitating ferroptosis. Oxidative stress further impairs granulosa cell function by reducing antioxidant capacity, steroidogenesis, and follicle-stimulating hormone receptor expression, contributing to abnormal follicular development (33).

Ferroptosis exerts a double-edged effect in endometriosis: while it damages ovarian granulosa cells, oocytes, and embryos—disrupting oocyte maturation, decreasing oocyte retrieval rates, and impairing embryonic development—it also promotes ectopic lesion growth through downstream signaling pathways that enhance angiogenesis and inflammation (34). Notably, differential susceptibility to ferroptosis exists between cell types: endometriotic cells acquire resistance through upregulation of GPx4 and GSH, whereas granulosa cells remain vulnerable due to decreased antioxidant defenses (35, 36).

Therapeutically, ferroptosis inducers can promote lipid ROS accumulation and endometriotic cell death but may exacerbate granulosa cell damage, while ferroptosis inhibitors protect granulosa cells against oxidative damage. Additionally, strategies targeting iron overload, such as iron chelators, and antioxidants have shown promise in ameliorating granulosa cell ferroptosis and improving reproductive outcomes.

### 2.4 Genetic, epigenetic, and molecular alterations

Genetic factors contribute to endometriosis-associated infertility, with studies highlighting polymorphisms in antioxidant enzyme genes. For example, variant genotypes of GPX1 Pro198Leu, CAT-262C>T, GSTM1 null allele, and combined GSTM1-GSTT1 null genotype are significantly associated with increased risk of endometriosis-related infertility, likely through disrupted oxidative stress regulation. Additionally, integration of gene expression data has identified 269 differentially expressed programmed cell death-related genes (DPGs) in endometriosis, with 17 showing causal associations and three (TNFSF12, AP3M1, PDK2) emerging as potential diagnostic biomarkers (37).

Epigenetic dysregulation plays a central role, involving DNA methylation, histone modifications, and non-coding RNAs. DNA methyltransferases (DNMT1, DNMT3A, DNMT3B) exhibit differential expression, while ten-eleven translocation methylcytosine dioxygenases (TET1-3) are downregulated in ectopic lesions and eutopic stromal cells, influencing 5-hydroxymethylcytosine levels and epithelial-mesenchymal transition. Methyl-CpG-binding domain protein MBD2 is decreased, potentially preventing repression of methylated genes. Genome-wide analyses reveal thousands of differentially methylated sites affecting steroid hormone signaling and inflammation pathways (38).

Histone modifiers are also dysregulated: histone deacetylases (HDAC1-3, SIRT1), methyltransferases (EZH2, MLL1), and demethylases (LSD1) alter chromatin states, contributing to progesterone resistance, inflammation, and fibrosis. MicroRNAs (miRNAs) regulate epigenetic enzymes post-transcriptionally; e.g., miR-148a modulates DNMT1 under hypoxia (38).

Chromatin remodeler ARID1A, critical for endometrial receptivity, is downregulated in eutopic endometrium of women with endometriosis, likely via epigenetic/transcriptional mechanisms rather than mutations. ARID1A directly binds the Foxa2 promoter to regulate its transcription, essential for endometrial gland function. Epithelial-specific Arid1a deletion in mice preserves gland number but reduces FOXA2 and gland-specific genes (Lif, Spink3, Cxcl15), causing implantation and decidualization defects through disrupted LIF-STAT3-EGR1 signaling. This leads to non-receptive endometrium with increased epithelial and decreased stromal proliferation, and exogenous LIF fails to rescue implantation, indicating additional ARID1A-dependent defects (35).

Transcription factor BCL6 is upregulated in endometriotic lesions and correlates with disease severity and poor IVF outcomes. It recruits corepressor complexes to repress target genes, and co-localizes with SIRT1 to repress GLI1, a mediator of progesterone action, linking BCL6 to progesterone resistance. BCL6 is regulated by IL6/STAT3 signaling and miRNAs, integrating inflammatory and epigenetic mechanisms (39).

### 2.5 Microbiome and metabolic influences

Emerging evidence highlights the intricate roles of reproductive tract and gut microbiome dysbiosis, alongside metabolic and coagulation system perturbations, in the pathogenesis of endometriosis and associated infertility.

The female genital tract (FGT) microbiome, typically dominated by Lactobacillus species in healthy individuals, exhibits significant dysbiosis in endometriosis. Endometriotic patients frequently show reduced Lactobacillus abundance and increased colonization by opportunistic pathogens such as Gardnerella, Prevotella, Streptococcus, and Enterococcus. This dysbiosis disrupts immune homeostasis by elevating proinflammatory cytokines and compromising immunosurveillance, thereby promoting ectopic lesion survival and growth. The “Bacterial Contamination Theory” proposes that such dysbiosis introduces lipopolysaccharides (LPS) into the uterine environment, which activate pattern recognition receptors through binding to the TLR4/MD2 complex, triggering MyD88-dependent signaling that phosphorylates IκB and liberates NF-κB for nuclear translocation, ultimately inducing chronic inflammatory cascades and further exacerbating endometriotic pathology. Clinically, endometrial microbiome profiles correlate with reproductive outcomes: a Lactobacillus-dominated (LD) endometrium (>90% Lactobacillus) associates with improved implantation and pregnancy rates in assisted reproductive technology (ART), whereas non-Lactobacillus-dominated (NLD) states predict poorer outcomes (40).

Gut microbiome dysbiosis similarly contributes to endometriosis pathogenesis through multiple mechanisms. In murine models, the presence of endometriotic lesions alters the Firmicutes/Bacteroidetes ratio, a key indicator of dysbiosis (41). This dysregulation impairs immune clearance of ectopic endometrial fragments, allowing lesion establishment and growth. Gut-derived LPS, a major component of Gram-negative bacterial cell walls, activates the TLR4/MyD88/NF-κB pathway, driving proinflammatory cytokine release and promoting adhesion, invasion, and angiogenesis of endometriotic lesions. Additionally, the gut microbiome modulates estrogen metabolism via the estrobolome: bacterial β-glucuronidase activity converts conjugated estrogen to bioactive forms, increasing circulating estrogen levels that fuel lesion growth. Altered β-glucuronidase-producing species are consistently identified in endometriosis patients. Microbial metabolites such as short-chain fatty acids (SCFAs), particularly butyrate, exert protective effects by enhancing regulatory T cell differentiation and intestinal barrier integrity, with butyrate treatment reducing lesion size in murine models (42).

Coagulation system factors, though less extensively studied in this context, intersect with microbiome-driven inflammation. Cytokine-mediated inflammation and altered macrophage function, linked to microbiota changes, may contribute to coagulation system dysregulation, influencing disease progression and reproductive outcomes. However, direct evidence for specific coagulation factors like ADAMTS13 or vWF remains limited and requires further investigation.

Therapeutic modulation of the microbiome shows promise: antibiotics targeting dysbiotic bacteria and fecal microbiota transfer (FMT) reduce lesion growth in preclinical models, while probiotics restoring Lactobacillus dominance improve NK cell activity and reproductive outcomes. Beyond the microbiome, other metabolic influences such as smoking and alcohol consumption can also impact reproductive function through altered hormone profiles, disrupted oviductal contractility, and impaired endometrial receptivity, further contributing to infertility in endometriosis (43).

## 3 Clinical impact of endometriosis on female fertility

**Figure 2:**
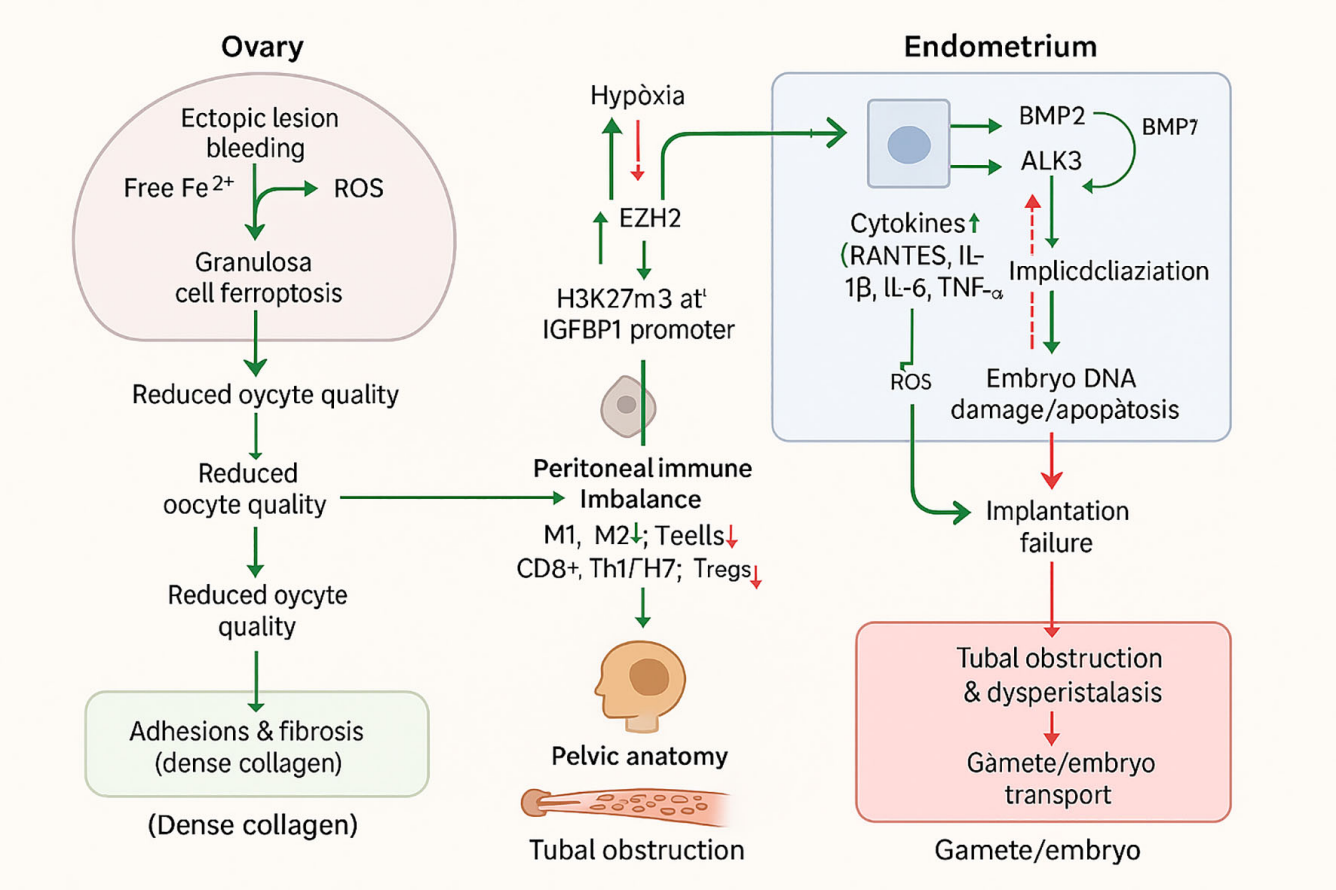
Clinical impact of endometriosis on female fertility.

Endometriosis exerts a profound and multifaceted impact on female fertility, affecting nearly every stage of the reproductive process—from ovarian function and oocyte quality to endometrial receptivity, tubal patency, and overall pelvic anatomy. Understanding these clinical manifestations is critical for guiding diagnostic strategies, selecting appropriate treatments, and optimizing reproductive outcomes for affected women (**Figure 2**).

### 3.1 Ovarian reserve and oocyte quality

Ovarian reserve and oocyte competence are significantly compromised in women with endometriosis, with ovarian endometriomas and the broader disease process playing pivotal roles. Ovarian endometriomas per se impair ovarian reserve, as evidenced by decreased serum anti-Müllerian hormone (AMH) levels, reduced antral follicle count (AFC), and histopathological findings of decreased follicular density and vascular abnormalities in the ovarian cortex surrounding these cysts, with up to 16% of cases exhibiting complete absence of follicles. Surgical excision of ovarian endometriomas further exacerbates this impairment by frequently causing unintentional removal of healthy ovarian tissue, leading to a sustained decline in serum AMH levels for at least 6–9 months and diminished ovarian response to controlled ovarian hyperstimulation (COH), particularly when cyst size exceeds 4 cm (44).

In terms of oocyte yield and maturity, ovarian endometriosis consistently reduces oocyte yield (mean difference [MD] −1.22) and the number of mature oocytes (MD −2.24). Meta-analyses of IVF/ICSI studies confirm that women with endometriosis have a significantly reduced number of oocytes retrieved per cycle and a lower percentage of mature oocytes, with a significant increase in follicle atresia observed in the affected ovary; this reduction is evident not only in follicles in close proximity to endometriomas but also in distal follicles of the same ovary (45).

The pathophysiological mechanisms underlying these impairments involve an altered follicular environment characterized by increased inflammation and oxidative stress. Iron overload from periodic bleeding of ectopic endometriotic lesions induces oxidative stress via free iron-mediated Fenton reactions, which freely diffuses into adjacent granulosa cells. This, combined with reduced antioxidants such as glutathione and glutathione peroxidase (GPx), increases granulosa cell susceptibility to ferroptosis—an iron- and reactive oxygen species (ROS)-dependent programmed cell death—ultimately impairing oocyte maturation and competence. Additionally, granulosa cells in women with endometriosis exhibit apoptosis, mitochondrial dysfunction, disrupted steroid hormone production, and increased inflammatory markers, further compromising follicle health (15).

These changes contribute to significant oocyte quality defects. Morphologically, oocytes from women with endometriosis frequently exhibit zona pellucida hardening, spindle disruption, nuclear anomalies, increased cytoplasmic granularity, and impaired cumulus-oocyte complex (COC) expansion. Functionally, ROS-induced DNA damage, reduced mitochondrial content, and a higher proportion of abnormal mitochondria have been observed, collectively diminishing oocyte competence. Severe endometriosis (ASRM stage III and IV) particularly influences all stages of reproduction, from folliculogenesis and oocyte quality to fertilization and embryo development, highlighting the broad impact of disease severity (2). Notably, while ovarian stimulation for IVF does not significantly worsen endometrioma size or ovarian reserve metrics, the underlying disease process remains a critical determinant of reduced oocyte quantity and quality (46).

### 3.2 Endometrial receptivity and implantation failure

The endometrium must be receptive for successful embryo implantation to take place. In endometriosis, impaired endometrial receptivity arises from interconnected mechanisms involving defective decidualization, altered immune cell populations, and a disrupted endometrial environment, collectively contributing to implantation failure.

Defective decidualization is a central feature, primarily driven by progesterone resistance and aberrant cell signaling in the eutopic endometrium (47). Endometriosis is an estrogen-dependent condition with dysregulated steroid hormone pathways, leading to progesterone resistance that impairs decidualization (48). Epigenetic mechanisms further exacerbate this: hypoxia, a key pathological process in endometriosis, upregulates histone methyltransferase EZH2, which catalyzes H3K27Me3 modification at the IGFBP1 promoter, suppressing this critical decidual marker and directly impairing decidualization (49). Additionally, loss of endometrial ARID1A disrupts the LIF-STAT3-EGR1 pathway, diminishing LIF expression – a cytokine necessary for implantation and decidualization – leading to a non-receptive endometrium characterized by altered epithelial and stromal proliferation (35). Bone morphogenetic protein (BMP) signaling, integral to endometrial remodeling, is also dysregulated: BMP2 promotes decidualization via ALK3-mediated upregulation of genes like WNT4 and IGFBP3, while BMP7 inhibits this process, and their imbalance contributes to defective decidualization (50).

Altered immune cell populations further disrupt endometrial receptivity. Endometriosis is associated with increased activated pelvic macrophages, T-lymphocytes, and natural killer (NK) cells in the peritoneal environment, driving an inflammatory cascade (10). Specifically, increased pro-inflammatory M1 macrophages and decreased M2 macrophages disrupt the cytokine milieu required for stromal decidualization, while endometrial macrophages exhibit defective phagocytic capacity and pro-inflammatory phenotypes. Uterine NK (uNK) cells show increased numbers but decreased expression of activating receptors like NKp46, indicating functional impairment. T cell populations are also imbalanced, with increased cytotoxic CD8+ T cells, pro-inflammatory Th1/Th17 subsets, and decreased regulatory T cells (Tregs), disturbing immune tolerance (51). These immune alterations are accompanied by elevated peritoneal fluid cytokines (RANTES, IL-1β, IL-6, TNF-α) and oxidative stress from reactive oxygen species, inducing DNA damage and apoptosis in embryos.

The endometrial environment is further compromised by hormonal asynchrony between the endometrium and embryo, resulting from fluctuations in serum progesterone and estradiol levels. Ovarian stimulation may exacerbate this by expanding the implantation window and increasing estrogen levels, potentially lowering pregnancy rates. Notably, deferred frozen-thawed embryo transfer (FET) may restore synchrony and reset the uterine environment, with studies reporting higher live birth rates and lower miscarriage rates compared to fresh transfer, though clinical routine use requires caution due to heterogeneity in evidence. However, conflicting findings exist: a transcriptomic study using the endometrial receptivity array (ERA) test found no difference in receptivity gene signatures during the implantation window between endometriosis patients and controls, and a sibling oocyte study showed no difference in implantation rates in severe endometriosis patients undergoing IVF, suggesting context-dependent effects. Emerging evidence suggests interventions like PPD may improve the endometrial environment by upregulating progesterone receptors, downregulating estrogen receptors, and normalizing immune cell function – including reducing pro-inflammatory cytokines (IL-12, IFN-γ) in macrophages and enhancing decidual NK cell-mediated immune tolerance and angiogenesis (52).

### 3.3 Tubal and anatomical factors

Anatomical distortions caused by adhesions and fibrosis represent key contributors to infertility in endometriosis, leading to mechanical obstruction and impaired pelvic anatomy relevant to reproductive function. Extensive pelvic adhesions can result in cul-de-sac obliteration, defined by Sampson (1922) as “extensive adhesions in the cul-de-sac, obliterating its lower portion and uniting the cervix or the lower portion of the uterus to the rectum, with adenoma of the endometrial type invading the cervical and the uterine tissue”, which compromises sperm passage and tubal function. Deep rectovaginal endometriotic nodules, histologically characterized as “adenomyomas consisting of smooth muscle hyperplasia with active glandular epithelium and scanty stroma”, correlate with significant pelvic adhesions and structural distortion. These nodules often coexist with uterine adenomyosis, with external uterine adenomyosis detected in over 97% of women with deep posterior endometriotic nodules ≥3 cm; such adenomyotic lesions can invade posterior uterine and cervical walls, extending into the rectovaginal space and digestive tract to cause mechanical obstruction (53).

Tubal involvement further contributes to infertility, as endometriotic lesions in the fallopian tube subserosal layer are strongly associated with reproductive impairment. Magnetic Resonance Imaging (MRI) may reveal T1-weighted hyperintensity within dilated fallopian tubes, a finding suggestive of endometriosis and potential structural compromise. Adhesions can also obliterate normal pelvic angles and induce abnormal uterine flexion, while nodularity and thickening of pelvic ligaments (uterosacral ligaments) with fibrotic reactions further disrupt pelvic anatomy (54). Fibrosis and smooth muscle proliferation are prominent in both adenomyotic and deep endometriotic lesions, forming dense collagen fibers that reinforce adhesions and structural alteration.

Mechanically, these changes impair fertility through multiple pathways: extensive adhesions can occlude the tubal ostium and embed ovaries, blocking sperm passage and oocyte release. Uterotubal dysperistalsis, a dysfunction in tubal motility, may further disturb gamete and embryo transport (11). In severe cases, anatomical damage to ovaries (including impacts on ovarian reserve) and fallopian tubes directly blocks gamete and embryo transport (55). Experimental baboon models demonstrate that nodular lesions can invade bowel muscularis and surrounding tissues, illustrating mechanical disruption relevant to tubal and pelvic anatomy.

Clinically, surgical laparoscopy aims to restore normal pelvic anatomy, though its benefit is most evident in minimal/mild disease. However, severely distorted pelvic anatomy increases surgical risk, making assisted reproductive technologies (ART) like IVF preferable in such scenarios (56). GnRH agonists may temporarily improve distorted anatomy and adhesions by reducing disease burden, potentially enhancing oocyte release or transport. Despite these interventions, the presence of adhesions, tubal blockage, and structural distortion remains a critical barrier to fertility in endometriosis.

### 3.4 Disruption of folliculogenesis and oocyte function by the endometriotic microenvironment

The endometriotic microenvironment is characterized by elevated levels of reactive oxygen species (ROS), iron overload, pro-inflammatory cytokines, and altered immune cell infiltration. These factors collectively contribute to a “toxic” milieu that compromises ovarian function at multiple levels. Iron-induced oxidative stress and ferroptosis in granulosa cells impair steroidogenesis, reduce mitochondrial function, and disrupt antioxidant defenses, leading to impaired folliculogenesis and oocyte maturation. Studies have shown that follicular fluid from women with endometriosis contains increased ROS and lipid peroxidation byproducts, which correlate with lower oocyte quality and fertilization rates (57).

Additionally, the persistent inflammatory environment alters the expression of genes involved in cumulus expansion, ovulation, and corpus luteum formation. Aberrant secretion of interleukins (e.g., IL-1β, IL-6) and TNF-α may impair ovulatory cascade signaling, while elevated prostaglandins and dysregulated angiogenesis can interfere with follicle rupture and luteal function. Furthermore, diminished levels of growth factors such as IGF-1 and GDF-9 within the follicular microenvironment may further contribute to poor oocyte competence and suboptimal embryo development (58, 59).

These disruptions highlight the multifactorial impact of the altered ovarian niche in endometriosis, offering a rationale for targeted antioxidant, anti-inflammatory, or ferroptosis-modulating therapies aimed at restoring follicular health and reproductive potential.

## 4 Current therapeutic strategies addressing endometriosis-associated infertility

### 4.1 Surgical management

Laparoscopic surgery serves as a cornerstone in the surgical management of endometriosis-associated infertility, aiming to remove visible lesions and restore pelvic anatomy to improve reproductive outcomes. Techniques include ablation and excision, with evidence indicating that compared to diagnostic laparoscopy alone, laparoscopic surgery probably increases viable intrauterine pregnancy rates in women with minimal to mild endometriosis (OR 1.89, 95% CI 1.25 to 2.86; 3 RCTs, 528 participants; moderate quality evidence). Advanced minimally invasive approaches such as robotic laparoscopy have further enhanced surgical precision and anatomical visualization, reducing postoperative complications and recovery time while effectively removing complex deep endometriosis lesions with preserved fertility potential (29, 52).

Discoid excision, a conservative surgical technique, is particularly relevant for managing colorectal deep endometriosis (DE) and has demonstrated favorable fertility outcomes. In a study of 49 patients desiring pregnancy, 51% (25/49) achieved pregnancy after discoid excision, with a high spontaneous pregnancy rate of 60% (15/25) and a live birth rate of 75% (12/16) among spontaneous conceptions. This approach is mainly indicated for lesions involving less than 90° of the bowel circumference and ≤3 cm in length (with double discoid excision feasible for larger lesions ~5 cm), aiming to reduce morbidity compared to segmental resection while maintaining similar recurrence rates. When performed by experienced surgeons in expert centers, discoid excision is considered safe, with postoperative complication rates of 24.5% (mostly minor Clavien-Dindo grades I-II) and no severe complications like rectovaginal fistula or anastomotic leakage reported (60). However, the impact of laparoscopic surgery on ovarian reserve remains an area requiring further investigation, as current evidence is insufficient to draw definitive conclusions, highlighting the need for high-quality RCTs with standardized outcome reporting.

### 4.2 Hormonal medical treatments

Hormonal medical treatments for endometriosis-associated infertility target the endocrine pathogenetic mechanisms of the disease, aiming to block menstruation through inhibition of the hypothalamus-pituitary-ovary (HPO) axis or induction of pseudodecidualization with consequent amenorrhea, thereby impairing the progression of endometriotic implants (6). These treatments include GnRH agonists, GnRH antagonists, progestins, oral contraceptives, and emerging selective receptor modulators.

GnRH agonists (goserelin, leuprolide, nafarelin, buserelin, triptorelin) downregulate pituitary GnRH receptors, leading to hypoestrogenism and amenorrhea, which results in regression of endometriotic lesions and improvement in pain symptoms. However, they are associated with hypoestrogenic side effects that can be mitigated with add-back therapy. In contrast, GnRH antagonists competitively inhibit GnRH receptors without causing an initial flare-up, offering rapid onset of action. Oral GnRH antagonists such as elagolix induce dose-dependent suppression of LH, FSH, and estradiol levels, reducing pain while minimizing severe hypoestrogenism. Their principal advantages include dose-dependent estrogen suppression, fast reversibility of hormone secretion after treatment cessation, oral delivery, and avoidance of the flare-up effect. Ongoing trials are evaluating other oral GnRH antagonists like relugolix and linzagolix.

Progestins, considered first-line hormonal therapies, act by decreasing FSH and LH secretion, inducing anovulation and amenorrhea, promoting endometrial pseudodecidualization, and inhibiting inflammation, angiogenesis, and endometriotic cell apoptosis. Key examples include dienogest (DNG), a 19-nortestosterone derivative approved for endometriosis, which effectively reduces pain and lesion size with good tolerability and minimal impact on bone mineral density; norethindrone acetate (NETA), FDA-approved for pain relief with potential androgenic side effects but effective and well-tolerated at low doses for long-term use; and medroxyprogesterone acetate (MPA), including depot formulations, which is effective but associated with bone mineral density loss with prolonged use. According to major international guidelines, low-dose combined hormonal contraceptives (CHCs) and progestogens are standard first-line treatments for symptomatic endometriosis, effective in approximately two-thirds of symptomatic women (61).

Combined oral contraceptives (COCs) are commonly used off-label to suppress ovarian function and reduce symptoms, though evidence supporting their effectiveness is less robust, with about 50% of patients reporting partial or no symptom improvement. Emerging hormonal agents include selective progesterone receptor modulators (SPRMs) such as ulipristal acetate and mifepristone, which have shown promising results in ameliorating endometriosis-associated pain; however, their safety profile regarding potential liver toxicity and progesterone receptor-associated endometrial changes (PAEC) in endometriotic foci has not been proven with sufficient evidence. Selective estrogen receptor modulators (SERMs) like raloxifene have demonstrated variable effects, with some reducing lesion size in preclinical models but limited clinical applications due to scarce and low-quality evidence. Aromatase inhibitors, which inhibit local estrogen synthesis in endometriotic tissue and can reduce pain, are recommended only for resistant cases in combination with other hormonal agents due to adverse effects and limited clinical data.

Safety considerations for hormonal treatments include potential cancer risks. While no conclusive evidence links hormonal fertility treatments to increased breast, colon, cervical, or endometrial cancer risk, ovarian cancer risk may be modestly increased, potentially confounded by underlying conditions such as endometriosis or nulliparity. Borderline ovarian tumors (BOTs) have a weakly increased incidence among treated women, with progesterone use associated with higher BOT risk compared to clomiphene citrate (CC) or gonadotropins. Prolonged use of CC (>10 cycles) may elevate breast cancer risk. These findings emphasize the importance of counseling patients about possible small increased risks, particularly regarding ovarian cancer and BOTs (62).

### 4.3 Assisted reproductive technologies

Assisted Reproductive Technology (ART) has evolved from being primarily indicated as second-line treatment or for cases involving male factor infertility to assuming a more prominent role in managing endometriosis-associated infertility, driven by technological advancements, delayed childbearing trends, and improved clinical and laboratory techniques. The combined approach of surgery followed by ART has been shown to enhance pregnancy chances in infertile women with endometriosis, though pelvic surgery for endometriosis—particularly for ovarian endometriomas—carries risks of iatrogenic damage, including ovarian reserve loss, adhesion formation, and ischemic injury.

For intrauterine insemination (IUI), women with stage I/II endometriosis may benefit from IUI combined with controlled ovarian hyperstimulation (COH), as this strategy increases live birth rates; following surgery, IUI performed within six months can yield pregnancy rates comparable to those observed in cases of unexplained infertility. However, a large multicenter cohort study identified endometriosis as a risk factor for IUI treatment failure, and while combining IUI with ovarian stimulation (using clomiphene citrate or gonadotropins) improved outcomes, results were not stratified by disease stage. Notably, patients with minimal/mild endometriosis-associated infertility achieve lower success rates with stimulation and IUI compared to women with unexplained infertility, but ablation of minimal/mild endometriosis normalizes clinical pregnancy rates per cycle and cumulative birth rates, indicating that endometriosis exerts a reversible detrimental effect on fertility (11).

*In vitro* fertilization (IVF), including intracytoplasmic sperm injection (ICSI), is recommended for moderate-to-severe endometriosis, particularly in cases with ovarian endometriomas larger than 3 cm and/or deep infiltrating endometriosis, even when tubal patency and semen parameters are normal. IVF outcomes vary by disease stage: while less advanced stages show success rates similar to other infertility causes, advanced stages are associated with lower fertilization, implantation, and clinical pregnancy rates. The primary limiting factor for ART success in endometriosis patients is ovarian response to stimulation, as previous surgery and pelvic adhesions can reduce ovarian response and oocyte retrieval (9). A key debate regarding IVF outcomes centers on the relative contributions of oocyte quality versus endometrial receptivity. A study comparing donor oocyte recipient cycles with autologous IVF cycles in women with endometriosis found no significant difference in live birth rates (LBR), suggesting that impaired endometrial receptivity, rather than oocyte quality, is the predominant factor contributing to reduced LBR in these patients (63).

Tailored ART strategies are critical to optimizing outcomes in endometriosis patients. Pre-treatment with GnRH agonists for 3–6 months prior to ovarian stimulation may improve ART outcomes by reducing pelvic inflammation. Antagonist protocols are preferred due to their ability to facilitate dual stimulation cycles, enable safer triggering, and reduce the risk of ovarian hyperstimulation syndrome, thereby optimizing oocyte yield. For women with reduced ovarian reserve due to endometriosis, segmented or double stimulation protocols can help increase the number of oocytes retrieved. Additionally, controlled ovarian hyperstimulation (COH) for ART does not increase endometriosis recurrence rates compared to women not undergoing COH after surgery. Fertility preservation, particularly oocyte vitrification, should be considered in selected cases at risk of ovarian reserve compromise, such as those with bilateral ovarian endometriomas, recurrent disease, or prior surgery. While IVF is not recommended as first-line treatment for infertility in general, it should be considered for women with endometriosis who are 38 years or older.

### 4.4 Supportive and adjunctive therapies

Supportive and adjunctive therapies play a complementary role in addressing endometriosis-associated infertility by targeting underlying pathophysiological mechanisms such as oxidative stress, immune dysregulation, and pain. Among these, antioxidants and micronutrients have garnered attention as potential modifiable interventions. The Priority Setting Partnership for Infertility has identified investigating the usefulness of nutraceuticals in improving reproductive potential as a key research priority, emphasizing the need for well-conducted studies into diet and nutraceuticals to explore their role in managing infertility; this aligns with the broader goal of identifying modifiable risk factors and assessing whether treating these factors improves outcomes (64).

Immunomodulators, including agents targeting pro-inflammatory pathways, are another focus of supportive therapy. Pentoxifylline, an immunomodulator and anti-inflammatory agent, has been specifically investigated for endometriosis-associated infertility and pain management. Its rationale lies in modulating immune mechanisms implicated in endometriosis pathophysiology, such as inhibition of TNF-α and reduction of inflammatory activation in immune cells. However, a Cochrane systematic review encompassing five randomized controlled trials (415 women) found insufficient very low-quality evidence to support its effectiveness in improving clinical pregnancy rate (RR 1.38, 95% CI 0.91 to 2.10), live birth rate, overall pain, miscarriage rate (Peto OR 1.99, 95% CI 0.20 to 19.37), or endometriosis recurrence (RR 0.84, 95% CI 0.30 to 2.36), highlighting the need for higher-quality trials (65).

Novel devices for pain and symptom management offer ultralow-invasive options that may preserve ovulatory function, a critical consideration for reproductive-aged women. The Angel Touch device (AT-04), a portable magnetic fields irradiation device utilizing mixed alternative magnetic fields at 2 kHz and 83.3 MHz, has shown promise in preclinical and early clinical studies. Its pain control mechanism involves regulating nerve growth factors, locally inhibiting inflammatory cytokines, and activating the descending inhibitory system. A pilot study in five women with endometriosis-related dysmenorrhea reported significant improvement in dysmenorrhea and reduced endometriotic cyst size without adverse events. Currently, an ongoing phase III, multicenter, randomized, sham-controlled, double-blind trial is evaluating AT-04’s efficacy and safety for endometriosis-related pain (dysmenorrhea, dyspareunia, chronic pelvic pain), with the primary outcome being the change in Numeric Rating Scale score at 16 weeks (36).

### 4.5 Disease heterogeneity and subtype-specific therapeutic strategies

Endometriosis exhibits significant clinical and biological heterogeneity, encompassing diverse anatomical subtypes—superficial peritoneal endometriosis (SPE), ovarian endometriomas (OMA), and deep infiltrating endometriosis (DIE)—each with distinct pathophysiological features, symptom profiles, and fertility implications (13). This heterogeneity complicates diagnosis and treatment selection, underscoring the need for precision medicine approaches tailored to disease subtype and individual patient characteristics (**Table 1**).

**Table 1:** Clinical features and precision treatment strategies for subtypes of endometriosis-associated infertility.

Endometriosis subtypes	Precise treatment strategy	Reference
Superficial peritoneal ectropion (SPE)	1. Drug priority: low-dose combined oral contraceptives (COCs) or danone progesterone to inhibit lesion activity and inflammation; 2. Minimally invasive intervention: for those who are ineffective with drugs or the diameter of lesions>1cm, laparoscopic lesion ablation (bipolar electrocoagulation or laser ablation) was performed to avoid excessive damage to peritoneal blood vessels during operation; 3. Fertility management: Try to conceive actively within 3–6 months after surgery, and improve the conception rate during the period of inflammation relief	Bonavina et al. (13), Chen et al (22), Gülden et al (29), Laura et al (61)
Ovarian endometrioma (OMA)	1. Surgical strategy: For cysts with diameter <4 cm, ultrasound-guided puncture and sclerotherapy (such as povidone-oligoglycol) were performed; for cysts with diameter ≥4 cm, laparoscopic “cyst stripping + tourniquet method” was used, and low-power bipolar electrocoagulation was used to stop bleeding during operation to protect ovarian tissue; 2. Postoperative management: serum AMH≥1.2ng/mL try natural conception or intrauterine artificial insemination (IUI);AMH<1.2ng/mL or directly perform IVF for advanced age (> 35 years old); 3. Adjuvant therapy: GnRH agonists (e.g., leuprolide) combined with anti-addition therapy were used in the short term (1–3 months) after surgery to suppress residual lesions	Rafael et al. (15), Yangshuo et al (34), Gustavo et al (44), Hiroshi et al (45), Phillips et al
Deep Infiltrating Endometriosis (DIE)	1. Multidisciplinary surgery: Pancreatic resection was performed for intestinal infiltration range <90° and circumferential cases (postoperative pregnancy rate reached 51%); For extensive infiltration (e.g., intestinal circumference>90° or ureteral obstruction), segmental bowel resection was performed in combination with gastrointestinal surgery, and pregnancy was attempted again 6–12 months after surgery; 2. Postoperative maintenance: use of dienogestrel or GnRH antagonist for 3–6 months to inhibit residual lesions and improve endometrial receptivity; 3. Assisted reproduction: For those with unobstructed fallopian tubes but poor endometrial receptivity, frozen embryo transfer (FET) was performed after progesterone pretreatment; for those with blocked fallopian tubes, IVF was directly performed	Lai et al (52), Jacques et al (53), Karen et al (54), Yohann et al (60), Kiwita et al

Superficial peritoneal lesions, often associated with cyclic pain and subtle anatomical disruption, may respond well to first-line hormonal therapies, particularly combined oral contraceptives and progestins. In contrast, ovarian endometriomas are strongly linked to diminished ovarian reserve and compromised oocyte quality, necessitating cautious surgical intervention and consideration of fertility preservation strategies such as oocyte vitrification. For patients with DIE, characterized by fibrotic, deeply invasive lesions often involving bowel, bladder, or uterosacral ligaments, fertility impairment results from both mechanical distortion and an inflammatory microenvironment. These cases frequently benefit from specialized conservative surgery by expert teams, sometimes followed by ART to optimize pregnancy outcomes.

Molecular and immune profiling further reveal subtype-specific differences. For instance, studies demonstrate distinct gene expression patterns and immune cell infiltrates between eutopic and ectopic tissues, and across lesion locations, suggesting variable responsiveness to immunomodulatory or microbiome-targeted therapies (27). Incorporating such biomarkers into clinical decision-making may enable stratification of patients for personalized interventions, including targeted hormonal suppression, immunotherapy (CGRP/RAMP1 blockade), or ferroptosis modulation.

Future therapeutic frameworks should thus integrate phenotypic, molecular, and patient-specific factors to guide individualized treatment. Subtype-directed care holds particular promise in infertility management, where aligning surgical, medical, and ART strategies to endometriosis phenotype can significantly improve reproductive outcomes while minimizing iatrogenic risks.

## 5 Emerging diagnostic biomarkers and predictive tools

### 5.1 Genetic and transcriptomic biomarkers

Genetic and transcriptomic biomarkers, particularly epigenetic regulators, have emerged as critical indicators for predicting endometriosis and associated infertility risks by modulating endometrial receptivity, decidualization, and implantation processes. Epigenetic mechanisms, encompassing histone modifications, chromatin remodeling, DNA methylation, and non-coding RNA regulation, are increasingly recognized as central to the pathophysiology of endometriosis-related infertility.

Histone-modifying enzymes represent key epigenetic markers. The histone methyltransferase EZH2 and its repressive mark H3K27Me3 are upregulated in endometrial stromal cells (ESCs) of endometriosis patients, contributing to defective decidualization. Hypoxia, a pathological feature of endometriosis, stabilizes EZH2 mRNA by reducing m^6^A RNA methylation through increased ALKBH5 (m^6^A demethylase) and decreased YTHDF2 (m^6^A reader), thereby enhancing EZH2 protein levels. EZH2 represses IGFBP1 (a decidualization marker) by increasing H3K27Me3 at the IGFBP1 promoter, impairing decidualization, while EZH2 knockdown or conditional deletion in mouse ESCs rescues decidualization defects and improves fertility (49). Additionally, histone deacetylases (HDAC1, HDAC2, HDAC3, SIRT1) are dysregulated in endometriosis, linking to inflammation, progesterone resistance, and fibrosis, further exacerbating infertility.

Chromatin remodeling factors also play a pivotal role. ARID1A, a chromatin remodeling protein, exhibits reduced expression in the eutopic endometrium of women with endometriosis, likely due to epigenetic regulation rather than genetic mutations. Uterine-specific deletion of Arid1a compromises gland development, diminishes Foxa2 and Lif expression, and disrupts the LIF-STAT3-EGR1 pathway, leading to implantation failure and subfertility. ARID1A directly binds the Foxa2 promoter to regulate its transcription, and reduced ARID1A correlates with decreased FOXA2 in both human and non-human primate endometriosis models (35).

DNA methylation modifiers, including DNA methyltransferases (DNMT1, DNMT3A, DNMT3B) and ten-eleven translocation (TET) methylcytosine dioxygenases (TET1, TET2, TET3), show altered expression in endometriosis, though findings may vary due to sample heterogeneity (cell types, menstrual cycle phases). For example, TET1 levels are decreased in the eutopic endometrium of infertile women with endometriosis, contributing to dysregulated DNA methylation patterns. Genome-wide DNA methylation studies reveal thousands of differentially methylated CpG sites in endometriotic lesions, affecting genes involved in hormone signaling (GATA family, progesterone receptor), immune regulation, and cell identity—critical for endometrial receptivity (38).

Transcriptomic biomarkers, particularly exosomal non-coding RNAs, are emerging as predictive tools. Exosomes derived from eutopic and ectopic endometrial cells in endometriosis patients exhibit altered profiles of miRNAs (miR-22-3p, miR-320a, miR-17, miR-106a), lncRNAs (LINC00998, NEAT1, PVT1), and circRNAs. These exosomal cargos regulate key implantation-related genes (HOXA10, LIF), inflammation, angiogenesis, and fibrosis, linking transcriptomic dysregulation to endometriosis pathophysiology and infertility (66).

### 5.2 Immune and inflammatory markers

Chronic inflammation and immune dysfunction are hallmark features of endometriosis, contributing to disease development and progression, with evidence from frequent co-occurrence with autoimmune diseases. The immune microenvironment in endometriosis is characterized by altered immune cell infiltration, such as increased proportions of macrophages M1 and M2, monocytes, CD4 memory resting T cells, and activated mast cells in ectopic tissues compared to eutopic endometrium. This immune dysregulation is accompanied by a pro-inflammatory milieu, driven by cytokines, immune checkpoint molecules, and inflammatory mediators, which are increasingly recognized as potential diagnostic biomarkers.

Pro-inflammatory cytokines play a central role in the pathophysiology of endometriosis, with elevated levels observed in both local (peritoneal fluid, ectopic lesions) and systemic (serum) compartments. Key cytokines implicated include IL-6, IL-1β, TNF-α, IL-8, and IL-17, which contribute to lesion attachment, proliferation, pain, impaired embryo development, and endometrial receptivity. Among these, TNF-α is highlighted as a critical driver, with elevated levels in serum and peritoneal fluid of patients with active lesions, and its altered balance with Th1/Th2 cytokines linked to embryo toxicity and endometrial dysfunction (25). Additionally, IL-16, derived from ectopic endometrial T cells via iron overload-induced caspase-3-GSDME-mediated pyroptosis, has emerged as a novel pro-inflammatory mediator; its levels are elevated in cystic fluid and serum of patients with ovarian endometriosis, correlating with inflammation markers and disease progression, suggesting diagnostic potential (67).

Immune checkpoint molecules, which regulate immune tolerance, are also dysregulated in endometriosis. For instance, increased exhausted PD1+ natural killer (NK) cells have been reported in advanced stages of the disease, indicating a role for immune checkpoint dysregulation in disease progression and potential as a diagnostic target.

Beyond cytokines and checkpoint molecules, other inflammatory mediators and pathways contribute to the pro-inflammatory microenvironment. Reactive oxygen species (ROS) and dysregulated iron homeostasis promote oxidative stress and immune dysregulation through activation of the NF-κB pathway, impairing macrophage phagocytic activity and contributing to resistance to ferroptosis (68). Complement component C3 production and activation of complement and coagulation cascades, as highlighted by single-gene Gene Set Enrichment Analysis of immune-related biomarkers (CXCL12, PDGFRL, AGTR1, PTGER3, S1PR1), further support the involvement of inflammatory pathways in disease pathogenesis (69).

While several immune and inflammatory markers show promise, their clinical reliability remains insufficient. Integrated approaches, such as transcriptomic and bioinformatics analyses, have identified key immune cell-related genes (CXCL12, PDGFRL, AGTR1, PTGER3, S1PR1) with robust diagnostic potential, and emerging strategies like miRNA signatures and immunophenotyping of menstrual effluent may enhance diagnostic accuracy. Further validation of these markers, particularly their specificity and sensitivity across disease stages, is needed to translate them into clinical practice.

### 5.3 Microbiome-based and coagulation biomarkers

Microbiome profiling and coagulation factors have emerged as promising non-invasive diagnostic candidates for endometriosis, offering potential insights into disease pathogenesis and clinical management. While microbiome-based biomarkers are an area of growing interest, recent advances in understanding coagulation factors have provided substantial evidence for their diagnostic utility. A key study investigating coagulation factors employed a two-sample Mendelian randomization (MR) approach using large-scale genome-wide association study (GWAS) summary statistics from the UK Biobank (4354 cases, 217,500 controls) and FinnGen (8288 cases, 68,969 controls) cohorts to explore causal associations between 11 coagulation factors and endometriosis risk. These factors, including vWF (von Willebrand factor), ADAMTS13, aPTT, FVIII, FXI, FVII, FX, ETP, PAI-1, protein C, and plasmin, were grouped by their roles in platelet adhesion, intrinsic and extrinsic pathways, common pathways, and fibrin dissociation.

Results from this study demonstrated a strong negative causal effect of genetically predicted plasma ADAMTS13 levels on endometriosis risk (protective factor), consistent across both cohorts and meta-analysis; conversely, plasma vWF levels showed a positive causal association with endometriosis risk (risk factor). Further MR analyses on endometriosis sub-phenotypes revealed that ADAMTS13 had a negative causal association with ovarian, pelvic peritoneum, and uterine endometriosis, while vWF exhibited positive causal effects on ovarian and pelvic peritoneum endometriosis. The biological functions of ADAMTS13 and vWF are interrelated, with ADAMTS13 regulating thrombotic activity by cleaving ultra-large vWF multimers; this balance influences hemostasis, inflammation modulation, angiogenesis, and tissue remodeling, which are key processes implicated in endometriosis pathogenesis. Collectively, these findings suggest that coagulation factors, specifically ADAMTS13 and vWF, represent promising non-invasive biomarkers and potential therapeutic targets in the diagnosis and management of endometriosis, highlighting their role in hypercoagulability and chronic inflammation observed in the disease (70).

While a wide range of potential biomarkers—including CA-125, various microRNAs, and immune-related molecules—have been identified for the diagnosis and monitoring of endometriosis, their clinical utility remains limited (71). Most currently proposed biomarkers suffer from inadequate sensitivity and specificity, particularly in early-stage disease or asymptomatic patients. Moreover, the majority of supporting studies are based on small, single-center cohorts and lack external validation, thereby restricting their generalizability.

Emerging approaches involving biomarker panels and multi-omics technologies (such as proteomics and miRNA expression profiling) offer promising avenues for improving diagnostic accuracy. However, these tools are still in the investigational stage, and their transition into clinical practice demands rigorous validation through large-scale, multicenter studies. Consequently, the application of these biomarkers in routine clinical settings should be approached with caution, and further research is needed to determine their true value in guiding diagnosis, treatment selection, and disease monitoring.

## 6 Novel therapeutic avenues and future directions

### 6.1 Immunotherapy and targeted molecular treatments

Recent studies have identified the calcitonin gene-related peptide (CGRP)/receptor activity modifying protein 1 (RAMP1) signaling axis as a critical player in endometriosis pathophysiology, with implications for both lesion development and pain generation (21). Endometriosis lesions from both human patients and mouse models express CGRP and its coreceptor RAMP1, highlighting the potential relevance of this pathway across species. Mechanistically, TRPV1+ nociceptors release CGRP, which acts through RAMP1 to shift macrophage polarization toward a pro-endometriosis phenotype (pro-endometriosis macrophages, PEMs); these PEMs exhibit impaired efferocytosis and promote endometrial cell growth. Single-cell RNA sequencing further revealed that macrophages, particularly small peritoneal macrophages (SPMs), express RAMP1 and are responsive to CGRP, thereby contributing to lesion growth and pain. Importantly, blocking CGRP/RAMP1 signaling using four FDA-approved drugs—anti-CGRP antibodies fremanezumab and galcanezumab, and RAMP1 antagonists rimegepant and ubrogepant—has been shown to reduce mechanical hyperalgesia, spontaneous pain, and lesion size in a mouse model of endometriosis. Additionally, targeted deletion of Ramp1 specifically in macrophages diminished pain-related behaviors and reduced lesion size and number, confirming the key role of CGRP/RAMP1-mediated nociceptor-to-macrophage communication as a novel avenue for immunotherapy and targeted molecular treatment in endometriosis, addressing both pain and lesion development.

### 6.2 Ferroptosis modulation and oxidative stress control

Iron overload resulting from periodic bleeding is a characteristic feature of endometriosis, which contributes to oxidative stress and plays a crucial role in the pathogenesis of endometriosis and its associated infertility. Ferroptosis, a form of programmed cell death dependent on iron and lipid reactive oxygen species (ROS), distinct from apoptosis, has been implicated in endometriosis. Notably, endometriotic cells exhibit resistance to ferroptosis, while granulosa cells remain highly susceptible to this process.

The ferroptosis pathway involves several critical mechanisms: iron uptake via transferrin receptor, ferritinophagy mediated by nuclear receptor coactivator 4 (NCOA4), iron export through ferroportin regulated by hepcidin, accumulation of lipid peroxides via the Fenton reaction, and antioxidant defense via glutathione peroxidase 4 (GPx4) and glutathione (GSH). In endometriotic cells, ferroptosis resistance mechanisms are up-regulated, including increased expression of GPx4 and GSH, and fibulin 1 (FBLN1) which inhibits ferroptosis, thereby promoting cell survival and lesion progression. Conversely, granulosa cells show reduced GPx4 expression and ferritinophagy-mediated iron overload, leading to heightened susceptibility to ferroptosis and impaired oocyte quality (45).

Therapeutic approaches targeting these pathways have been explored. Ferroptosis inducers, such as erastin, promote lipid ROS accumulation and selectively kill endometriotic cells in *in vitro* and animal models. On the other hand, ferroptosis inhibitors, including ferrostatin-1, iron chelators, and antioxidants, have shown potential in protecting granulosa cells from ferroptosis and oxidative damage, though clinical evidence supporting these strategies is currently lacking.

### 6.3 Microbiota manipulation and metabolic interventions

Microbiota manipulation and metabolic interventions represent emerging innovative therapeutic strategies in the management of endometriosis-associated infertility, with growing evidence supporting their potential through modulation of gut microbial communities and targeting of key metabolic pathways. Dietary approaches, such as the Mediterranean diet (MD), have been explored for their role in influencing these pathways, with studies highlighting associations between MD adherence and improvements in reproductive health outcomes.

In the context of endometriosis, an experimental study demonstrated that adherence to the MD over 5 months resulted in a significant reduction in general pain and improvement in overall condition among women with laparoscopically confirmed endometriosis-associated pain, although this study was limited by the absence of a control group and reliance on self-reported adherence. The beneficial effects of the MD are hypothesized to stem from its anti-inflammatory and antioxidant properties, attributed to components such as extra virgin olive oil, vegetables, fruit, and long-chain omega-3 fatty acids, which may mitigate endometriosis risk and symptoms, whereas trans-fats and high red meat intake have been linked to increased risk.

For infertility, multiple prospective cohort studies have reported associations between higher MD adherence and improved outcomes in assisted reproductive technologies (ART), including increased numbers of available embryos, fertilized oocytes, clinical pregnancy rates, and live births, with some studies finding no association with pregnancy loss. The antioxidant components of the MD—such as polyphenols, vitamins C and A, β-carotene, folate, and dietary fiber—may enhance reproductive tract antioxidant status, counteract oxidative stress, and protect against reactive oxygen species-related damage, thereby supporting fertility. Additionally, gut microbiota modulation by dietary fiber, a key MD component, is thought to influence immune system responses and oxidative stress, processes relevant to reproductive health conditions including infertility. Collectively, these findings support the role of fecal microbiota influences and metabolic pathways (oxidative stress, inflammation) as underlying mechanisms for the beneficial effects of the MD in reproductive health, highlighting microbiota manipulation and metabolic interventions as promising avenues for further exploration (72).

### 6.4 Integration of multidisciplinary and personalized approaches

The management of endometriosis-associated infertility is increasingly recognizing the necessity of individualized, multimodal care that integrates emerging scientific insights with patient-centered strategies. Enhancing understanding of the underlying mechanisms of menstruation-related disorders, including endometriosis, is pivotal to advancing personalized care, as highlighted in a comprehensive initiative that brought together investigators and stakeholders across multiple disciplines, including population health, public sectors, and patient-facing organizations. This collaborative approach, exemplified by the 2018 “Menstruation: Science and Society” meeting, emphasized incorporating the patient voice from the outset, aiming to bridge basic physiology with clinical challenges in diagnostics, treatment, and education (73).

A cornerstone of such personalized care is the inclusion of patient-reported outcome measures (PROMs) into electronic health records, which has the potential to transform comparative effectiveness research and enhance patient-centered management. Additionally, digital health tools, such as mobile health (mHealth) apps, offer innovative platforms for patient engagement and focused data analysis, facilitating direct communication of information to women and promoting their active involvement in their care. Standardization of terminology and data collection in menstrual health research is also critical, as it promotes consistency and enables the integration of diverse data sources to tailor interventions to individual patient needs.

Addressing broader societal implications, including stigma associated with menstruation and endometriosis, requires targeted health communication strategies accessible to groups with low literacy, ensuring that care is equitable and inclusive. Furthermore, involving women and advocacy groups in study design and dissemination shifts the focus toward optimizing overall women’s health, aligning with the increasing desire for fertility preservation and uterine health among women who may delay pregnancy. By fostering interdisciplinary collaboration across scientific, clinical, and social science disciplines, and engaging patient-facing organizations, innovative solutions can be developed to address the complex needs of women with endometriosis-associated infertility across the reproductive lifespan.

Despite the comprehensive synthesis of pathophysiological mechanisms and therapeutic strategies in endometriosis-associated infertility, current evidence is limited by methodological biases, including predominance of observational studies and insufficient RCTs. A considerable proportion of studies are observational in nature—such as retrospective cohort or case-control designs—rendering them prone to selection bias and confounding, thereby limiting causal inference. For instance, the widely cited study by Barri et al. employed a retrospective cohort design to assess surgical outcomes, yet lacked randomization or blinding, which diminishes the internal validity of the findings (29). Similarly, Gupta et al. noted that much of the evidence linking oxidative stress to infertility in endometriosis derives from small-scale or non-randomized studies (10). Moreover, high-quality randomized controlled trials (RCTs) remain scarce: a Cochrane review by Bafort et al. concluded that, although laparoscopic surgery may improve fertility in minimal-to-mild endometriosis, the evidence is based on only three RCTs with moderate methodological quality (12). This scarcity of rigorous trials hampers the development of standardized, evidence-based treatment algorithms. Future research should prioritize large-scale, multicenter RCTs with well-defined outcomes and standardized staging systems to enhance the reliability and generalizability of therapeutic recommendations.

## 7 Conclusion

Endometriosis-associated infertility results from complex interactions among hormonal, immune, inflammatory, oxidative, genetic, and microbiome-related factors. Key mechanisms include estrogen dominance with progesterone resistance, immune dysfunction, oxidative stress-induced ferroptosis, and disrupted endometrial receptivity. These processes collectively impair ovarian reserve, oocyte quality, implantation, and pelvic anatomy.

While current treatments—including surgery, hormonal therapy, and ART—offer varying success depending on disease stage and phenotype, emerging strategies such as immunotherapy, ferroptosis modulation, and microbiota-based interventions show promise. Advances in biomarker discovery may further enable non-invasive diagnosis and personalized therapy.

To improve outcomes, an integrated, patient-centered approach that combines mechanistic insights, individualized treatment, and digital health tools is essential. Ongoing research is critical to fully elucidate the disease and optimize fertility care.

## Data Availability

The original contributions presented in the study are included in the article/supplementary material. Further inquiries can be directed to the corresponding authors.
